# Assessing transpulmonary pressure via direct pleural manometry

**DOI:** 10.1186/s13613-020-00718-z

**Published:** 2020-08-03

**Authors:** Christopher Rugg, Stefan Schmid, Janett Kreutziger, Mathias Ströhle

**Affiliations:** Department of General and Surgical Critical Care Medicine, Medical University of Innsbruck, Anichstrasse 35, 6020 Innsbruck, Austria

To the Editor,

With great interest we read the multicentre, prospective study on PEEP titration conducted in ARDS patients by Bergez et al. [1]. PEEP was set on the basis of two cleverly devised protocols—one referring to plateau the other to transpulmonary pressure (*P*_L_). Both were able to improve oxygenation compared to baseline settings with the latter slightly increasing possible over-distension. In accordance with an also recently published expert review by Gattinoni et al., they recognized diverse limitations and assumptions while using esophageal pressure (*P*_es_) as a surrogate for pleural pressure (*P*_pl_) [2]. Nonetheless, another animal and human cadaver study revealed that *P*_es_ accurately reflects *P*_pl_ in the mid to dependent lung [3]. Regarding studies on mechanical ventilation, direct measurement of *P*_pl_ is usually restricted to animal or cadaver studies and is typically described as not feasible in clinical practice [2]. In 2018, a detailed review by Zielinska-Krawczyk et al. revealed that regarding therapeutic thoracenteses literature on direct pleural manometry actually is quite copious [4]. Direct pleural manometry has been used for studying various pathophysiological aspects of thoracentesis including the safety of high-volume pleural fluid removal. Furthermore, a portable electronic manometer, easy to build and only using widely accessible elements has been described [5]. Therefore, a small-bore catheter, inserted into the pleural cavity was connected with a vascular pressure transducer via a 3-way stopcock. After referencing the pressure to the catheter insertion point into the chest, *P*_pl_ was precisely measured.

We would like to share our experience in performing such measurements in an ICU setting. From a total of over 600 patients per year in our department (23 beds, mixed ICU), over 100 receive pleural pigtail-catheters, mostly indicated for gas exchange-limiting pleural effusions. These patients are usually decompensated of heart or pulmonary failure, intubated and to some extent mechanically ventilated. Our anatomical landmark for catheter insertion is the inferior angle of the scapula, with the patient in lateral position. The catheter tip then usually lies dorsally in the middle to caudal section of the pleural cavity, with position controlled by chest radiography and sonography. Awareness of catheter tip position is fundamental for adequate interpretation of measured pressures, as is the fact that pleural surface pressure may differ from pleural fluid pressure [4]. Measurements can be intermittently displayed on our standard ICU monitor as a pressure curve for example, monitoring also negative values (Fig. 1). Noteworthy is that the units displayed are usually mmHg and one must convert to cmH_2_O. In so doing we are able to conduct reliable, real-time measurements of *P*_pl_ during the complete respiratory cycle, regardless of ventilation mode. Complications are scarce, include pneumo- or haematothorax and occur mainly during insertion or removal of the catheter.

Of course, in critically ill patients pleural effusions are not always present, but if they are, drainage with a small-bore catheter not only helps improve oxygenation, but also gives a possibility to easily and directly measure *P*_pl_ and thus titrate ventilator settings without further deployment of additional devices. This can be beneficial in settings of limited resources. Direct pleural manometry via thoracic pigtail-catheters can be seen as an alternative to rather than a replacement of esophageal measurements in a special subgroup of patients.

**Fig. 1:**
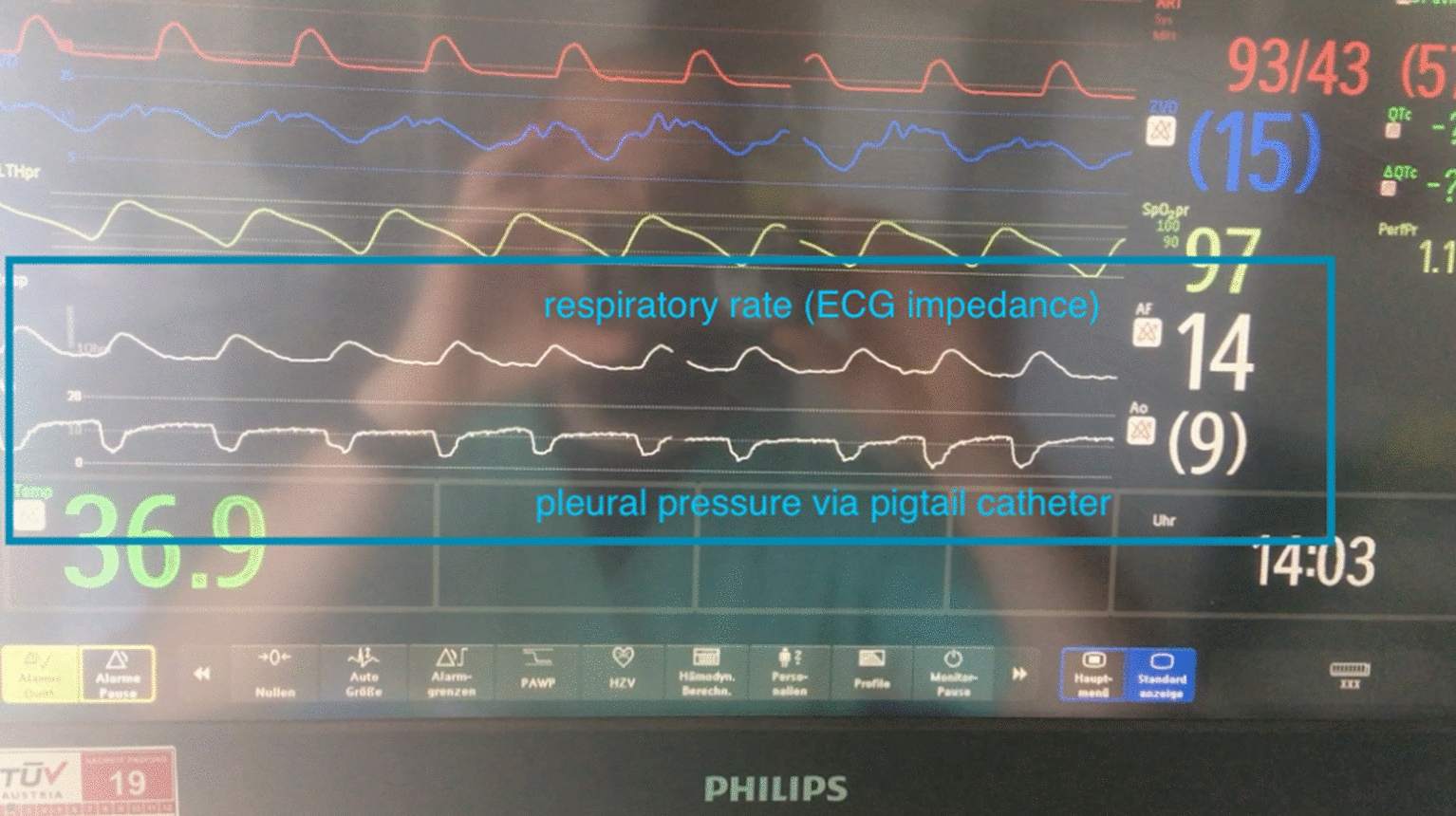
Direct pleural manometry of an intubated, spontaneously breathing patient in an ICU setting

## Data Availability

Not applicable.

## Abbreviations

*P*_L_: Transpulmonary pressure
*P*_es_: Esophageal pressure
*P*_pl_: Pleural pressure
